# Exploratory Analysis of MicroRNA (miRNA) as a Prognostic and Predictive Biomarker in Locally Advanced and Metastatic Gastric Cancer

**DOI:** 10.7759/cureus.103209

**Published:** 2026-02-08

**Authors:** Charles L, Yadav Nisha, Babu Vishva, Esha Jafa, Vikram Kate, Rajesh N G, Sandhiya Selvarajan, Smita Kayal, Biswajit Dubashi, Prasanth Penumadu, Prasanth Ganesan

**Affiliations:** 1 Medical Oncology, Jawaharlal Institute of Postgraduate Medical Education and Research, Puducherry, IND; 2 Medical Oncology, King George's Medical University, Lucknow, IND; 3 General Surgery, Jawaharlal Institute of Postgraduate Medical Education and Research, Puducherry, IND; 4 Pathology, Jawaharlal Institute of Postgraduate Medical Education and Research, Puducherry, IND; 5 Clinical Pharmacology, Jawaharlal Institute of Postgraduate Medical Education and Research, Puducherry, IND; 6 Surgical Oncology, Jawaharlal Institute of Postgraduate Medical Education and Research, Puducherry, IND

**Keywords:** gastric cancer tissue, mir-25, mir-27a, mir-29, mirna expression, qrt-pcr, survival, twofold expression

## Abstract

Background and purpose

Emerging evidence suggests that microRNAs (miRNAs) can function as oncogenes or tumor suppressors, playing an important role in pathogenesis, treatment response, and survival outcomes. This study aims to identify miRNAs as prognostic and predictive biomarkers in locally advanced and metastatic gastric cancer.

Materials and methods

This study was a prospective exploratory study of patients with gastric cancer from April 2018 to October 2022. Tissues of 50 paired locally advanced and metastatic gastric cancer patients were examined for the expression level of oncomiRs miR-21, miR-200b, miR-27a, miR-93, miR-18a, miR-25, miR-210, and miR-29c and tumor suppressor miR-204 using quantitative real-time reverse-transcription polymerase chain reaction (qRT-PCR), and the relevant association with clinical factors was analyzed.

Results

The expression of miR-29c, miR-27a, and miR-25 was differentially regulated into low and high based on the twofold change. Patients with low miR-29c expression demonstrated an increased propensity for metastasis (P = 0.03). Downregulation of miR-25 was significantly associated with the absence of peritoneal involvement (P < 0.0001) and liver metastasis (P < 0.005). High expression of miR-27a was associated with a better response to chemotherapy (P = 0.04). It was observed that the median follow-up duration was 7.98 months (range, 0.60-46.93 months). Notably, patients in the low miR-27a expression group had significantly better overall survival compared to the high expression group (12.03 (5.69-18.37) months vs 4.90 (3.47-6.33) months; P = 0.02).

Conclusion

The differentially regulated miRNAs, namely, miR-29c, miR-27a, and miR-25, may be used as predictive and prognostic biomarkers in advanced gastric cancer which require further validation.

## Introduction

As per the Global Cancer Statistics 2020, gastric cancer is the fifth most common cancer and the fourth leading cause of cancer-related mortality [1]. The incidence and progression of gastric cancer are regulated by multiple factors, including genetic predisposition, environmental exposures, dietary habits, lifestyle choices, microbial and viral infections (such as *Helicobacter pylori* and Epstein-Barr virus), as well as genetic and epigenetic alterations. Molecules like microRNAs (miRNAs) and long non-coding RNAs (lncRNAs) serve as promising prognostic and predictive biomarkers; however, their roles remain poorly understood and warrant further investigation [2-4].

miRNAs are a class of new short endogenous non-coding RNAs with 18-23 nucleotides that are conserved across a wide range of species and negatively influence gene expression. miRNA maturation typically involves a number of processes. miRNAs negatively regulate gene expression in various biological processes, including differentiation, morphogenesis, proliferation, and apoptosis [5].

In gastric cancer, dysregulated miRNAs have been identified in tumor tissues, and their aberrant expression patterns have been associated with clinicopathological characteristics, such as tumor stage, lymph node involvement, and patient survival. These findings suggest that miRNAs may serve as valuable diagnostic and prognostic biomarkers in gastric cancer [6]. Understanding the role of miRNAs in gastric cancer tissue could have significant clinical implications. It may provide insights into the underlying molecular mechanisms driving gastric cancer pathogenesis and help identify potential therapeutic targets [7]. Additionally, the identification of miRNAs that are differentially expressed in gastric cancer tissues could lead to the development of miRNA-based therapies or the use of miRNAs as predictive biomarkers for treatment response [8]. In the present study, we examined the expression of eight oncomiRs, namely, miR-21, miR-200b, miR-27a, miR-93, miR-18a, miR-25, miR-210, and miR-29c, and one tumor suppressor miR-204 in 50 pairs of gastric cancer tissues and matched adjacent non-tumorous tissues. Besides, we further investigated their prognostic relationships with clinicopathological features (e.g., metastasis, peritoneal/liver involvement) and survival and predictive associations with chemotherapy response in advanced gastric cancer patients. Staging of the tumors was done using the American Joint Committee on Cancer (AJCC) TNM 7th Edition criteria [9].

This article was previously presented as an oral presentation as Exploratory Analysis of miRNA as Prognostic and Predictive Biomarker in Locally Advanced and Metastatic Gastric Cancer at the International Conference on Emerging Concepts in Biotechnological Innovations, SRM Institute of Science and Technology, Chengalpattu, India, on March 29 and 30, 2023.

## Materials and methods

Study population

This prospective exploratory study was conducted at Jawaharlal Institute of Postgraduate Medical Education and Research, a regional cancer center in Puducherry, from April 2018 to October 2022 and was approved by the institute's Institutional Ethics Committee (approval number: JIP/IEC/2017/304). A total of 50 paired gastric tumor tissues and adjacent normal tissues were collected from patients suspected of having gastric cancer who underwent routine diagnostic endoscopy. Written informed consent was obtained from all individuals prior to obtaining fresh tumor and non-tumor samples through forceps biopsy in the Department of Surgery, Jawaharlal Institute of Postgraduate Medical Education and Research, India. Only patients with a confirmed histological diagnosis of adenocarcinoma were included in the study. Demographic, clinical, histopathological, chemotherapy, and survival data were collected using a standardized proforma. 

Sample size

This is an exploratory study to understand the role of various miRNAs in advanced and metastatic gastric cancer. Sample size for this study was calculated using the OpenEpi software version 3 (Dean AG, Sullivan KM, Soe MM. OpenEpi: Open Source Epidemiologic Statistics for Public Health, www.OpenEpi.com, updated 2013/04/06), taking the sensitivity of miRNA-21 and relative precision of 12%, with 95% confidence interval. The minimum sample size required was calculated as 41, and adding 10% lost to follow-up, the total estimated sample size was 50 patients.

Inclusion criteria

Patients with newly diagnosed, histologically confirmed, locally advanced, or metastatic gastric carcinoma planned for first-line chemotherapy were included. Both male and female patients aged 18 years and above were eligible.

Exclusion criteria

Patients previously diagnosed with gastric carcinoma and who had received chemotherapy outside the study center were not included. Those who had not completed a minimum of three cycles of chemotherapy were excluded. Patients without documented post-chemotherapy response assessment were also excluded from the analysis.

Treatment

In the perioperative setting, 17 patients received chemotherapy. Epirubicin, oxaliplatin, and capecitabine (EOX) was the most commonly prescribed regimen, administered to 16 patients, while one patient received capecitabine monotherapy. In the adjuvant setting, one patient received capecitabine and oxaliplatin (CapOx), and two patients received EOX. In the palliative setting, the most common regimen was EOX, administered to 18 patients, followed by capecitabine monotherapy in two patients, CapOx in one patient, docetaxel plus carboplatin in one patient, and nanoparticle paclitaxel plus carboplatin in one patient. Supportive care was provided to six patients. In addition, eight patients experienced treatment delays, and dose modifications were required in 10 patients across the entire cohort.

Sample collection and RNA extraction

Tumor and adjacent non-tumor tissue samples were collected via forceps biopsy in the Department of General Surgery at Jawaharlal Institute of Postgraduate Medical Education and Research, after obtaining informed consent from all participating individuals. Non-tumor gastric specimens located more than 2 cm from the tumor were used as normal gastric mucosa. For each case, two tissue samples from the tumor and two from the normal site were obtained. Each freshly collected tissue sample was immediately placed in a tube containing RNAlater solution and preserved at -80°C until further processing. Total RNA was extracted from both gastric cancer tissues and adjacent normal gastric mucosal tissues using the miRNeasy Mini Kit (cat. no. 217004; Qiagen), as per the manufacturer's instructions. The concentration of total RNA was determined by measuring the absorbance ratio at 260 and 280 nm using a BioSpectrometer (Eppendorf, Stevenage, UK). The extracted RNA was stored at -80°C until use. Complementary DNA (cDNA) was synthesized from 1 µg of extracted RNA using the miRCURY LNA RT Kit (cat. no. HB-2431; Qiagen).

Quantitative real-time reverse-transcription polymerase chain reaction (qRT-PCR)

miRNA expression was determined using Qiagen miRNA primer/probe sets (Hilden, Germany). All qPCR reactions were performed on the QuantStudio™ 5 Real-Time PCR System (Applied Biosystems, Thermo Fisher Scientific, Waltham, MA, USA). cDNA synthesized from extracted RNA was used as the template for qPCR, utilizing the miRCURY LNA miRNA Probe PCR kit (cat. no. 339350; Qiagen) following the manufacturer's protocol. The cycling parameters were as follows: initial denaturation at 95°C for two minutes, followed by 40 cycles of 95°C for five seconds and 56°C for 30 seconds. To evaluate the clinical relevance of miRNAs in gastric cancer, the expression of nine miRNAs was measured in our cohort of 50 gastric cancer tissues (T) and matched non-cancerous gastric tissues (N) using qRT-PCR. Differences in miRNA expression profiles between tumor and control tissues were assessed. Differentially regulated miRNAs (DRGs) were identified based on the 2−ΔΔCT method, with a threshold of a twofold change. DRGs were classified as having high or low expression using the mean ΔCT of the tumor as the cut-off. Additionally, the mean miRNA expression levels in gastric cancer tissues were used to stratify patients with advanced gastric cancer into two groups: high miRNA expression and low miRNA expression. SNORD (housekeeping gene) was used for data normalization. The details of miRNA are described in Table 1.

**Table 1:** List of miRNA, sequence, and mechanism of action

SI. no.	Name of miRNA	Sequence	Target gene and pathway	Mechanism of action	Expression
1	miR-21-3	(5'CAACACCAGUCGAUGGGCUGU)	RECK, PDCD4, PTEN	Cell proliferation, cell cycle, metastasis, apoptosis	Upregulated [10,11]
2	miR-200b-5p	(5'CAUCUUACUGGGCAGCAUUGGA)	ZEB1 and ZEB2	Cell proliferation, migration, invasion	Upregulated [12]
3	miR-27a-5p	(5'AGGGCUUAGCUGCUUGUGAGCA	PHB (prohibitin), ZBTB10, FOXO1	Cell proliferation, migration	Upregulated [13,14]
4	miR-18a-5p	(5'UAAGGUGCAUCUAGUGCAGAUAG)	PIAS3	Cell proliferation, apoptosis	Upregulated [15]
5	miR-93-5p	(5'CAAAGUGCUGUUCGUGCAGGUAG)	p57, p21	Cell proliferation, cell cycle	Upregulated [16]
6	miR-25-5p	(5'AGGCGGAGACUUGGGCAAUUG)	p57, p21	Cell proliferation, cell cycle	Upregulated [17]
7	miR-210-3p	(5'CUGUGCGUGUGACAGCGGCUGA)	Hif-1α	Cell proliferation, apoptosis, angiogenesis	Upregulated [18]
8	miR-29c-5p	(5’UGACCGAUUUCUCCUGGUGUUC)	CDK6, RCC2, PPIC	Apoptosis	Downregulated [7,16]
9	miR-204-5p	(5'UUCCCUUUGUCAUCCUAUGCCU)	Bcl-2	Apoptosis	Downregulated [19]

Statistical analysis

Statistical analyses were performed using IBM SPSS Statistics for Windows, V. 19.0 (IBM Corp., Armonk, NY, USA). Categorical data were expressed as frequencies and percentages, while continuous data were summarized using the mean with standard deviation (SD) and median with range. The chi-squared test was used to investigate the association between miRNA expression and clinicopathological variables. Additionally, survival analysis, focusing on overall survival (OS), was conducted using the Kaplan-Meier method.

## Results

Clinical characteristics of the study subjects

Demographic and clinicopathological characteristics of 50 gastric cancer patients are shown in Table 2. The study population included 31 (62%) males and 19 (38%) females, with eight (16%) of the participants aged ≤40 years, 26 (52%) aged >40-60 years, and 16 (32%) aged >60 years. A significant portion of patients (31, 62%) were underweight, while only four (8%) were overweight or obese, and 35 (70%) had an Eastern Cooperative Oncology Group (ECOG) performance status of 0-1. Comorbidities were present in 12 (24%) patients, with the most common being diabetes mellitus and hypertension (5, 41.6%), and symptoms included abdominal pain (32, 64%) and vomiting (30, 60%). In terms of disease staging, 22 (44%) had locally advanced disease, and 28 (56%) had metastatic disease, with a single metastasis found in 26 (93%) of metastatic cases. The most frequent histopathological type was intestinal (23, 60.5%), with the antropyloric region being the most commonly affected site (34, 68%).

**Table 2:** Demographic and clinical features of gastric cancer patients Age is represented as median (interquartile range). *Site of multiple metastasis (2): liver, nodal, lung (1);  bone, peritoneal, omental (1) ECOG: Eastern Cooperative Oncology Group; NACT: neoadjuvant chemotherapy; GEJ: gastroesophageal junction

Sl. no.	Variable	Total number	Category	N	(%)
1	Gender	50	Female	19	38
			Male	31	62
2	Age	50	≤40	8	16
			>40-60	26	52
			>60	16	32
3	Body mass index	50	Underweight	31	62
			Normal weight	14	28
			Overweight and obese	4	8
			Not available	1	2
4	ECOG	50	0-1	35	70
			2	11	22
			3-4	4	8
5	Comorbidity	50	Yes	12	24
6	Type of comorbidities	12	Diabetes mellitus	2	16.7
			Hypertension	2	16.7
			Diabetes mellitus and hypertension	5	41.6
			Coronary heart disease and hypertension	1	8.3
			Tuberculosis	2	16.7
7	Type of symptoms	50	Abdominal pain	32	64
			Vomiting	30	60
			Loss of weight	23	46
			Loss of appetite	19	38
			Melena	12	24
			Dyspepsia	2	4
			Abdominal distension	2	4
			Hematemesis	4	8
			Mass abdomen	1	2
8	Gastric outlet obstruction	50	Positive	13	26
9	TNM staging (early and locally advanced)				
	T status	22	T1-3	10	45.5
			T4a	7	31.8
			T4b	5	22.7
	N status	22	Nx	1	4.5
			N1	6	27.3
			N2	13	59.1
			N3	2	9.1
10	Stage	50	Locally advanced	22	44
			Metastasis	28	56
11	Metastasis	28	Single	26	92.9
			Multiple*	2	7.1
12	Site of single metastasis	26	Peritoneal	11	42.3
			Liver	9	34.6
			Nodal	4	15.4
			Bone	1	3.8
			Ovary	1	3.8
13	Histopathology subtype	38	Diffuse	15	39.5
			Intestinal	23	60.5
14	Site of tumor	50	GEJ/cardia	4	8
			Fundus and body	11	22
			Antrum and pylorus	34	68
			Linitis plastica	1	2
15	Treatment				
	Surgery	50	Yes	9	18
			No	41	82
	Chemotherapy	50	NACT	17	34
			Adjuvant	3	6
			Palliative	24	48
			No chemo	6	12
	Radiotherapy	-	-	-	-
16	Albumin	50	Median (range)	3.50 (2.0-4.0)	-

Differentially expressed miRNA in tissues of gastric cancer patients

The expression of miRNA-29, miRNA-27a, and miRNA-25 was upregulated in the gastric cancer tissue, while the expression of miRNA-200, miRNA-18a, miRNA-93, miRNA-210, miRNA-21, and miRNA-204 was downregulated in gastric cancer patients compared to the normal gastric tissue based on a twofold change as shown in Table 3. Further, the miRNA mean ΔCT of the tumor was taken to differentiate between low and high.

**Table 3:** List of miRNAs and fold changes between normal and tumor tissues

Sl. no.	miRNA	Gastric cancer 2−ΔCT (N = 50)	Adjacent normal 2−ΔCT (N = 50)	Fold change 2−ΔΔCT (N = 50)
1	miRNA-29	0.27 ± 0.46	0.17 ± 0.43	2.26 ± 3.23
2	miRNA-200	0.53 (-0.80, 1.49)	0.34 (-0.94, 0.94)	0.88 ± 0.92
3	miRNA-27a	-1.73 ± 0.86	-1.83 ± 0.87	2.39 ± 2.95
4	miRNA-18a	0.35 ± 0.42	0.29 ± 0.40	1.63 ± 1.49
5	miRNA-93	1.03 (0.16, 1.91)	1.01 (0.54, 1.95)	1.45 ± 1.18
6	miRNA-25	-1.34 ± 0.58	-1.48 ± 0.55	2.14 ± 2.49
7	miRNA-210	0.49 ± 0.49	0.57 ± 0.44	1.50 ± 2.11
8	miRNA-21	-0.33 ± 0.49	-0.04 ± 0.46	0.94 ± 1.17
9	miRNA-204	-0.80 ± 0.72	0.01 ± 0.54	0.55 ± 1.04

Association of clinicopathological features, treatment response, and survival with miRNA-29, miRNA-27a, and miRNA-25

A high expression of miRNA-29 (≥1.09) was observed in 28 (56%) patients, while a low expression (<1.09) was seen in 22 (44%). A statistically significant association was seen between miRNA-29 expression and diagnosis, with patients with low miRNA-29 expression having metastatic disease (16, 72.7%) compared to those with locally advanced disease (6, 27.3%) (P = 0.03). No significant associations were found between miRNA-29 expression and other variables such as gender, age, clinical T and N stages, metastatic site, peritoneal involvement, liver metastasis, histological subtypes (intestinal vs diffuse, signet ring vs non-signet ring), tumor location, or treatment response.

A high expression of miRNA-27a (≥5.8) was found in 17 (34%) patients, while a low expression (<5.8) was seen in 33 (66%). The analysis reveals a statistically significant association between miRNA-27a expression and treatment response (P= 0.048), with a higher proportion of patients with high miRNA-27a expression achieving a good responder (7, 87.5%) compared to those with low expression (1, 12.5%). Response assessment was done in 25 patients who completed six cycles. However, a higher percentage of patients with high miRNA-27a expression experienced a response to chemotherapy (10, 58.8%) compared to those with low expression (1, 12.5%) after chemotherapy. No significant associations were found between miRNA-27a expression and other clinical variables.

The miRNA-25 expression was high (≥4.45) in 25 (50%) of the patients, while the remaining 25 (50%) exhibited low expression (<4.45). The patients with downregulated miRNA-25 expression had an absence of both peritoneal involvement (P < 0.0001) and liver metastasis (P < 0.005). Specifically, 15 (60%) of the patients with high miRNA-25 expression had peritoneal involvement, compared to only one (4%) of those with low expression. On the other hand, nine (36%) of the patients with low miRNA-25 expression had no liver metastasis, in contrast to one (4%) of those with high expression. There were no significant associations between miRNA-25 expression and other factors, as shown in Table 4.

**Table 4:** Association of miRNA with clinicopathological features Treatment response: responder = partial response + stable disease; non-responder = progressive disease Age is represented as median (interquartile range). All the data have been represented as median (interquartile range). The chi-squared test was used to investigate the association between miRNA expression and clinicopathological variables. *Clinical TNM staging (cT and cN) was available only for patients with locally advanced disease (n = 22). ^#^Laurén's histological classification could not be determined for one patient who was diagnosed at an outside center, as it was not reported in the available records (n = 49). ^$^Among the study population, treatment response data were available for 25 patients (n = 25). LA: locally advanced; GEJ: gastroesophageal junction

Sl. no.	Variable		miRNA-29		P-value	miRNA-27a		P-value	miRNA-25	
			High (>1.09)	Low (<1.09)		High (>5.8)	Low (<5.8)		High (>4.45)	Low (<4.45)
			28 (56%)	22 (44%)		17 (34%)	33 (66%)		25 (50%)	25 (50%)
1	Gender (n = 50)	Female	8 (28.6)	11 (50)	0.12	7 (41.2)	12 (36.4)	0.37	8 (32)	11 (44)
		Male	20 (71.4)	11 (50)		10 (58.8)	21 (63.6)		17 (68)	14 (56)
2	Age (n = 50)	≤40	5 (17.9)	3 (13.6)	0.10	4 (23.5)	4 (12.5)	0.75	4 (16)	4 (16)
		<40-60	11 (39.3)	15 (68.2)		8 (47.1)	18 (54.2)		14 (56)	12 (48)
		>60	12 (42.8)	4 (18.2)		5 (29.4)	11 (33.3)		7 (28)	9 (36)
3	cT (n = 22)*	T1-3	8 (50)	2 (33.3)	0.48	4 (44.4)	6 (46.2)	0.93	4 (30.8)	6 (66.7)
		>T3	8 (50)	4 (66.7)		5 (55.6)	7 (53.8)		9 (69.2)	3 (33.3)
4	cN (n = 22)*	N1-2	14 (87.5)	6 (100)	0.36	9 (100)	11 (84.6)	0.49	12 (92.3)	8 (88.9)
		>N2	2 (12.5)	0 (0)		0 (0)	2 (15.4)		1 (7.7)	1 (11.1)
5	Diagnosis (n = 50)	LA	16 (57.1)	6 (27.3)	0.03	9 (52.9)	13 (39.4)	0.77	13 (52)	9 (36)
		Mets	12 (42.9)	16 (72.7)		8 (47.1)	20 (60.6)		12 (48)	16 (64)
6	Site (n = 28)	Single	10 (83.3)	16 (100)	0.17	7 (87.5)	19 (95)	0.48	10 (83.3)	16 (100)
		Multiple	2 (16.7)	0 (0)		1 (12.5)	1 (5)		2 (16.7)	0 (0)
7	Peritoneal vs non-peritoneal (n = 50)	No	22 (78.6)	16 (72.7)	0.63	10 (58.8)	24 (72.7)	0.31	10 (40)	24 (96)
		Yes	6 (21.4)	6 (27.3)		7 (41.2)	9 (27.3)		15 (60)	1 (4)
8	Liver vs no liver (n = 50)	Yes	5 (17.9)	5 (22.7)	0.66	3 (17.6)	7 (21.2)	0.24	9 (36)	1 (4)
		No	23 (82.1)	17 (77.3)		14 (82.4)	26 (78.8)		16 (64)	24 (96)
9	Intestine vs diffuse (n = 49)^#^	Intestine	19 (70.4)	11 (50)	0.23	12 (75)	18 (54.5)	0.38	15 (62.5)	15 (60)
		Diffuse	8 (29.6)	11 (50)		4 (25)	15 (45.5)		9 (37.5)	10 (40)
10	Signet vs no signet (n = 20)	No signet	1 (11.1)	0 (0)	0.45	1 (16.7)	0 (0)	0.30	9 (90)	10 (100)
		Signet	8 (88.9)	11 (100)		5 (83.3)	14 (100)		1 (10)	0 (0)
11	Site group (n = 50)	GEJ/cardia	2 (7.1)	2 (9.1)	0.38	1 (5.9)	3 (9.1)	0.79	3 (12)	1 (4)
		Fundus and body	4 (14.3)	7 (31.8)		3 (17.6)	8 (24.2)		6 (24)	5 (20)
		Antrum and pylorus	21 (75)	13 (59.1)		13 (76.5)	21 (63.6)		16 (64)	18 (72)
		Diffuse	1 (3.6)	0 (0)		0 (0)	1 (3)		0 (0)	1 (4)
12	Treatment response (n = 25)^$^	Responder	7 (50)	7 (63.7)	0.79	7 (87.5)	7 (41.2)	0.048	10 (71.4)	4 (36.4)
		Non-responder	7 (50)	4 (36.3)		1 (12.5)	10 (58.8)		4 (28.6)	7 (63.6)

Survival

**Figure 1:**
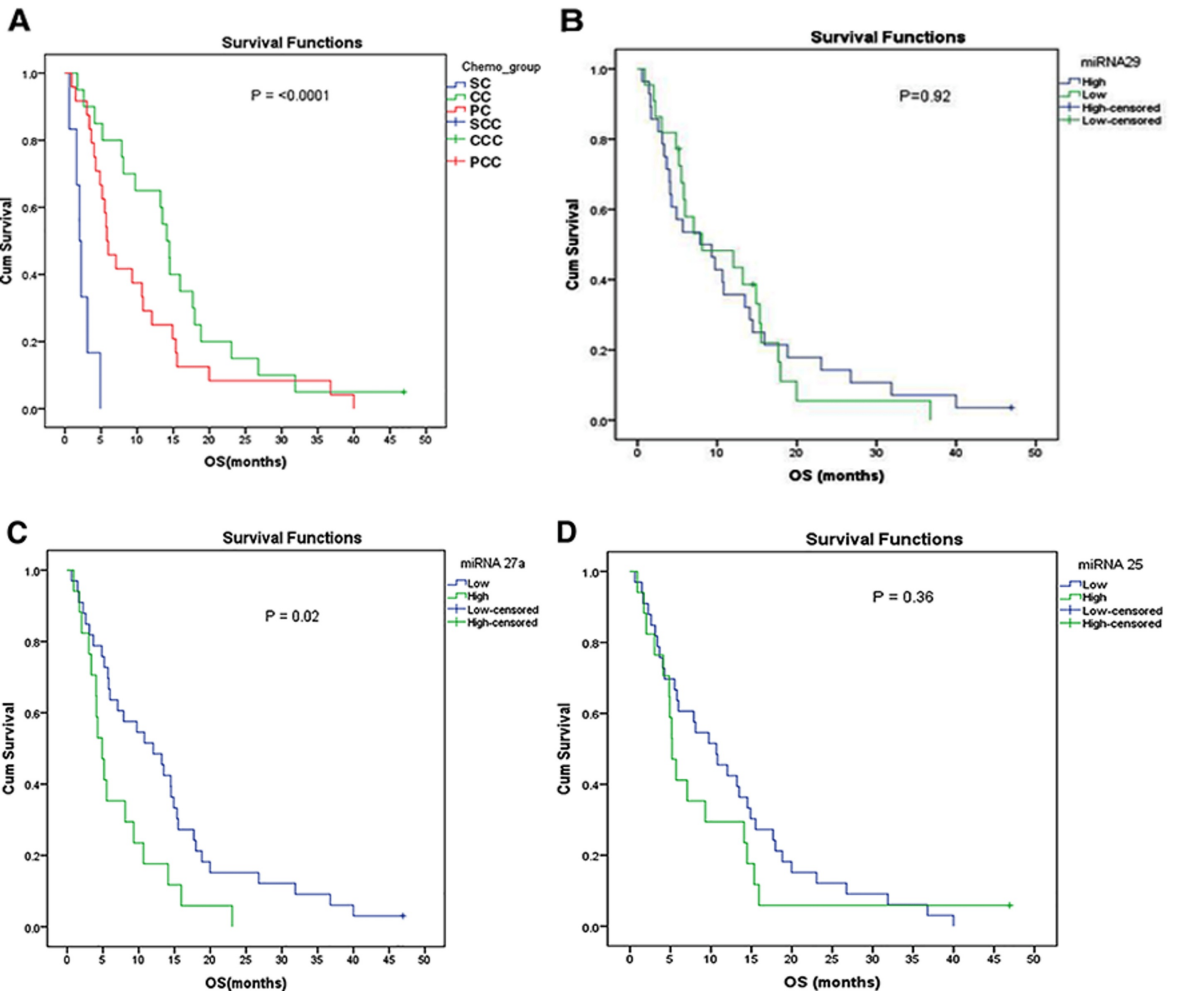
Overall survival of the study population stratified by miRNA group (A) Overall survival of the entire cohort. (B) Survival analysis of miRNA-29 between low and high expression. (C) Survival analysis of miRNA-27a between low and high expression. (D) Survival analysis of miRNA-25 between low and high expression. SC: supportive care; CC: curative chemotherapy; PC: palliative chemotherapy; SCC: supportive care censored; CCC: curative chemotherapy censored; PCC: palliative chemotherapy censored

The Kaplan-Meier analysis demonstrates significantly different OS among treatment groups (P < 0.0001). Patients treated with curative chemotherapy, OS of 14.1 months (11.98-16.22), showed the longest median survival, followed by palliative chemotherapy, OS of 5.8 months (3.92-7.68), and then supportive care as shown in Figure 1A. The median OS in miRNA-29 was 8 (2-14) months in the low expression group, and OS was eight (1-17) months in the highly expressed miRNA (P = 0.92) as shown in Figure 1B, which is not significant. The low expression miRNA-27a group had significantly better OS compared with the high expression group (12.03 (5.69-18.37) months vs 4.90 (3.47-6.33) months; P = 0.02) as shown in Figure 1C. The low expression miRNA-25 group was found to have better OS compared to the high expression group, but the difference was not statistically significant (10.67 (5.98-15.36) months vs 5.20 (4.12-6.28) months; P = 0.36) as shown in Figure 1D.

## Discussion

In recent years, research has demonstrated that abnormal miRNA expression contributes to the development and progression of gastric cancer by impacting the proliferation of tumor cells [20]. There are several studies reporting profiles of gastric cancer tissue miRNAs [21]. We found that miR-29c, miR-27a, and miR-25 increased significantly in gastric cancer. Our findings identified potential biomarkers that warrant further investigation across different gastric cancer cohorts. Moreover, this study demonstrated that miRNA expression levels fluctuated in tumor samples.

miR-27a

Numerous studies have demonstrated the aberrant expression of miR-27a in gastric cancer [14,22]. Ding et al. observed that the upregulation of miR-27a in gastric cancer tissues, compared to adjacent non-tumor tissues, and its expression level correlated with tumor size, invasion depth, and lymph node metastasis [23]. Wang et al. reported the upregulation of miRNA-27a in gastric cancer compared to normal tissues, which was observed in our study. He also reported that the inhibition of miRNA-27 leads to suppressed proliferation, invasion, and migration of gastric cancer cells by targeting RUNX1 [24]. Wu et al. found that high expression of miR-27a acts as a promoter of tumor progression by downregulating the SFRP1 gene, which leads to the significant upregulation of Wnt, p-β-catenin, and p-Wnt [25]. Similarly, Zhang et al. reported increased miR-27a expression in gastric cancer tissues and that its upregulation was associated with poor OS [14]. Zhou et al. analyzed the expression profiles of 20 pairs of gastric cancer tissues and their corresponding normal tissues. They found that both miR-27-3p and miR-27a-5p were significantly elevated in gastric cancer samples compared to adjacent non-tumor tissues, with average fold changes of 2 [22]. Similarly, we found that high expression of miR-27a is a prognostic biomarker in the OS of gastric cancer patients.

miR-29c

Several studies have reported the dysregulation of miR-29c in gastric cancer [26,27]. Wang et al. observed that miR-29a expression was significantly downregulated in gastric cancer tissues in comparison to adjacent non-tumor tissues. This decreased expression was found to be associated with advanced tumor stage and lymph node metastasis [28,29]. Tokumaru et al. observed low-expressed miR-29c in gastric cancer tissues and that its low expression correlated with poor OS [30]. As per Pei et al.'s study, miR-29a targets TET1, leading to its downregulation and subsequently promoting epithelial-mesenchymal transition (EMT) in breast cancer. Overall, these findings indicate that miR-29a functions as a tumor promoter by suppressing TET1, thereby enhancing cell proliferation and EMT in breast cancer [31]. miR-29c exhibits a dual function in cancer, both acting as a tumor suppressor and, in some contexts, potentially promoting oncogenic processes. miR-29c can promote EMT in certain contexts, leading to the increased migration and invasion of cancer cells. For example, in gastric cancer, miR-29c was found to target d-catenin (CTNND1), which plays a role in cell adhesion and metastatic signaling. The dysregulation of miR-29c can contribute to chemoresistance and promote aggressive tumor behavior [26]. The result of the current study did not find any significant association with clinical characteristics, except that patients with locally advanced cancer had lower expression of miR-29c compared to those with metastatic cancer (P = 0.03).

miR-25

Yang et al. demonstrated that miR-25 is highly expressed in gastric cancer cells and promotes cell proliferation while inhibiting apoptosis. miR-25 mediates these effects by targeting and suppressing EGR2, which is a tumor suppressor gene. These findings indicate that the miR-25/EGR2 axis plays a vital role in gastric cancer progression and may serve as a potential therapeutic target [17]. Larki et al. quantified the expression levels of miR-25 using qRT-PCR. The results indicated that miR-25 had a mean ΔCT value of 5.11 ± 4.24 in the gastric cancer group (n = 39), 5.96 ± 2.54 in the gastric dysplasia (GD) group (n = 33), and 4.25 ± 3.29 in the normal gastric (NG) group (n = 29). These values show promise in distinguishing early-stage gastric cancer from benign gastric diseases (P = 0.0003). The upregulation of miR-25 was associated with positive lymph node metastasis (P = 0.05) [32]. Zhao et al. suggested that miR-25 promotes gastric cancer growth and metastasis partly through RECK suppression. miR-25 is significantly overexpressed in gastric cancer tissues and cell lines, contributing to tumor progression by enhancing cell proliferation, migration, and invasion. miR-25 targets RECK, a tumor suppressor, to exert its oncogenic effects. Overexpression of RECK can counteract the tumor-promoting actions of miR-25 [33]. The result of the current study found that the downregulated miR-25 correlates with the absence of liver (P < 0.005) and peritoneal metastasis (P < 0.0001).

Limitations

This is an exploratory study with 50 paired tumor and matched normal gastric cancer tissues; the association between miRNA expression and response to chemotherapy, which was initially planned as part of this study, could not be included due to logistic constraints that limited the collection of post-chemotherapy biopsy samples. The findings need to be validated in independent, clinically uniform multicenter cohorts with larger sample sizes and multivariate analyses to establish clinical utility.

## Conclusions

The upregulation of the differentially regulated miRNAs, miR-29c, miR-25, and miR-27a, in gastric cancer tissues compared to normal gastric tissues was associated with poor survival in patients with locally advanced and metastatic gastric cancer. miRNAs are dysregulated in gastric cancer and play a significant role through the regulation of target genes involved in crucial cellular processes. miRNAs hold promise as a potential biomarker for a predictive and prognostic role in gastric cancer. Its dysregulated expression in gastric cancer tissue makes it an attractive candidate for detecting chemotherapy response as a predictive biomarker. Furthermore, targeting particular miRNAs may represent a promising therapeutic approach for gastric cancer treatment. However, further research is necessary to fully elucidate the precise molecular mechanisms underlying miRNAs in gastric cancer and to explore their therapeutic implications.
